# The c-Met receptor tyrosine kinase inhibitor MP470 radiosensitizes glioblastoma cells

**DOI:** 10.1186/1748-717X-4-69

**Published:** 2009-12-22

**Authors:** James W Welsh, Daruka Mahadevan, Ron Ellsworth, Laurence Cooke, David Bearss, Baldassarre Stea

**Affiliations:** 1 Departmentr of Radiation Oncology, The University of Texas M. D. Anderson Cancer Center, 1515 Holcombe Blvd., Houston, TX; USA; 2 Department of Medicine, University of Arizona Cancer Center, 1515 North Campbell Avenue, Tucson, AZ 85724-5024, USA; 3 University of Arizona College of Medicine, 1501 North Campbell Avenue, Tucson, AZ 85724-5024, USA; 4 SuperGen Inc, 4140 Dublin Boulevard, Dublin, CA 94568-7757, USA; 5 Department of Radiation Oncology, University of Arizona Cancer Center, 1515 North Campbell Avenue, Tucson, AZ 85724-5024, USA

## Abstract

**Purpose:**

Glioblastoma multiforme (GBM) is resistant to current cytotoxic therapies, in part because of enhanced DNA repair. Activation of the receptor tyrosine kinase c-Met has been shown to protect cancer cells from DNA damage. We hypothesized that inhibiting c-Met would decrease this protection and thus sensitize resistant tumor cells to the effects of radiation therapy.

**Materials and methods:**

Eight human GBM cell lines were screened for radiosensitivity to the small-molecule c-Met inhibitor MP470 with colony-count assays. Double-strand (ds) DNA breaks was quantified by using antibodies to gamma H2AX. Western blotting demonstrate expression of RAD51, glycogen synthase kinase (GSK)-3β, and other proteins. A murine xenograft tumor flank model was used for *in vivo *radiosensitization studies.

**Results:**

MP470 reduced c-Met phosphorylation and enhanced radiation-induced cell kill by 0.4 logs in SF767 cells. Cells pretreated with MP470 had more ds DNA damage than cells treated with radiation alone. Mechanistically, MP470 was shown to inhibit dsDNA break repair and increase apoptosis. MP470 influences various survival and DNA repair related proteins such as pAKT, RAD51 and GSK3β. *In vivo*, the addition of MP470 to radiation resulted in a tumor-growth-delay enhancement ratio of 2.9 over radiation alone and extended survival time.

**Conclusions:**

GBM is a disease site where radiation is often used to address both macroscopic and microscopic disease. Despite attempts at dose escalation outcomes remain poor. MP470, a potent small-molecule tyrosine kinase inhibitor of c-Met, radiosensitized several GBM cell lines both *in vitro *and *in vivo*, and may help to improve outcomes for patients with GBM.

## Introduction

The management of malignant gliomas continues to pose a difficult therapeutic challenge. Use of radiation therapy and chemotherapy after maximal tumor debulking can improve both local control and survival for some patients with malignant gliomas [1]. Unfortunately, despite adjuvant therapy nearly all patients with glioblastoma multiforme (GBM) will eventually develop tumor recurrence and die of the disease. Patterns-of-failure studies conducted after primary therapy for GBM have shown that 75%-90% of patients experience tumor recurrence within 2 cm of the resection margin [2]. Attempts to increase the local radiation dose have not led to any meaningful improvement in survival when tested in randomized trials [3]. The most likely cause of recurrence is believed to be intrinsic radioresistance, mediated in part by efficient DNA repair. This suggests that interventions aimed at modifying cellular resistance to radiation or chemotherapy may confer a survival benefit.

Hepatocyte growth factor (HGF) is a multifunctional heterodimeric protein typically produced by mesenchymal cells. Its pleiotropic activities are mediated through its cellular receptor, a transmembrane tyrosine kinase encoded by the proto-oncogene c-Met. In malignant cells, HGF has been shown to protect cells from death induced by a variety of DNA-damaging agents, including radiation and topoisomerase inhibitors [4]. Interestingly HGF/SF not only blocked DNA damage-induced apoptosis but also enhanced the rate of repair of DNA strand breaks [5]. HGF also functions as an autocrine or paracrine growth factor and activates a program of cell dissociation and motility coupled with increased protease production that has been shown to promote cellular invasion [6,7]. HGF and c-Met are co-expressed and often overexpressed in a broad spectrum of human solid tumors including lung, breast, and brain malignancies [7,8]. Therefore, the overexpression of c-Met by GBM cells suggests that blocking HGF or its receptor c-Met might be an attractive strategy when combined with conventional treatment for the treatment of GBM. A recent review of this approach indicates that several novel inhibitors of the tyrosine kinase activity of c-Met have been developed and tested as a single agent or in combination with cytoxic chemotherapy [9]. Although it has previously been shown that targeting HGF or c-Met expression using ribozyme radiosensitizers in GBM cells in vitro and xenograft tumor in vivo [10], demonstration of clinically useful inhibitors of the tyrosine kinase activity of c-Met combined with radiation have not been previously tested in GBM models.

In the work presented here, a novel inhibitor of c-Met tyrosine kinase, MP470 [11], was tested for its ability to radiosensitize GBM cells both *in vitro *and *in vivo*.

## Materials and methods

### Cell culture

All of the human GBM cell lines tested (SF763, SF268, SF295, SF126, SF188, SF767, U-87, and SF210) were obtained from the University of California, San Francisco, and maintained in Dulbecco's Modified Eagle Medium supplemented with 10% fetal calf serum and 1% penicillin-streptomycin [12]. Cells were incubated at 37°C in a 5% CO_2 _incubator. MP470 (SuperGen, Dublin, CA) was stored in the dark at 4°C until use, when it was dissolved in dimethyl sulfoxide and used at a final concentration of 5.0-10 *μ*M. The drug was added to cells 1 hour before irradiation unless otherwise specified. Control cells were treated with equal volumes of dimethylsulfoxide. A cobalt-60 teletherapy unit (Atomic Energy of Canada Limited Theratron-80) was used to irradiate the GBM cells at a dose rate of 2 Gy/min.

### Cell proliferation assay

The cytotoxicity of MP470 was assessed *in vitro *in all eight cell lines by using an MTS assay performed in a 96-well plate format. Cells were plated with a multichannel pipetter and MP470 (to a final concentration of 5 *μ*M) was added to triplicate wells 24-48 hours later, after which the plates were incubated for up to 4 days. The MTS assay was done with a CellTiter 96 AQ_ueous _Non-Radioactive Cell Proliferation Assay kit as per the manufactures recommendations (Cat #G5421, Promega, Madison, WI). The IC_50 _(the concentration that inhibited proliferation in 50% of the samples) was determined from standard curves.

### In vitro clonogenic assay

The eight human GBM cell lines were cultured as described above, harvested, counted, and seeded onto 60-mm petri dishes at specific cell densities. MP470 (5 *μ*M) was added 1 hour before the cells were irradiated with single doses ranging from 2 to 8 Gy, after which the cells were returned to a 37°C incubator and cultured for 14 days in the presence of the MP470 before fixation. Cells were fixed for 5 minutes with 3:1 methanol: acetic acid solution and stained for 5 minutes with 0.5% crystal violet (Sigma, St. Louis, MO) in methanol. Colonies were counted with a Colcount automated colony counter (Optronix, Milton Port, Oxford, UK) using the discrete colony mode. The surviving fraction was calculated as (mean colony counts)/(cells plated) × (plating efficiency), where plating efficiency was defined as (mean colony counts)/(cells plated for unirradiated controls). All experiments were done in duplicate in 3 independent experiments and averaged data points represent the means ± standard deviations (SD).

### Apoptosis assay

Near-confluent SF767 cells were pretreated with 5 *μ*M MP470 irradiated (8 Gy), and analyzed 4 hours later as follows. Briefly, after pretreatment with MP470 for 5 hours, cells were suspended in phosphate-buffered saline (PBS, made by dissolving 8 g NaCl, 0.2 g KCl, 1.44 g Na_2_HPO_4_, and 0.24 g KH_2_PO_4 _in 800 mL of H_2_O) (pH 7.2) containing acridine orange (1 *μ*g·mL^-1^) and RNAse A and then co-stained with 1 *μ*g·mL^-1 ^ethidium bromide (all reagents from Sigma); cells were then washed and examined under a fluorescence microscope (×150, Olympus, Center Valley, PA; exciting filter BG12, barrier filter 0-5.30). For quantitative analyses, 200 cells were counted and the percentages of necrotic and apoptotic cells calculated.

### γ-H2AX assay

Double-stranded (ds) DNA breaks lead to the formation of γ-H2AX, a unique histone complex. We used a γ-H2AX antibody (Upstate Cat #05-636, Millipore, Billerica, MA) to visualize dsDNA breaks as follows. Cells were plated in chamber slides, grown for 48 hours, and treated with 5 *μ*M MP470, one hour later, the cells were irradiated with 4 Gy and processed either 1 hour or 8 hours later. Cells were first fixed in 4% paraformaldehyde and incubated with the primary antibody against γ-H2AX. The primary antibody was then washed off, and a secondary antibody conjugated to fluorescein isothiocyanate (FITC) was added to the slides. DNA damage was visualized by using confocal microscopy. Median intensity of each cell was calculated using Photoshop and a 2 sided t-test was used to calculate the difference.

### Comet assay

dsDNA breaks were visualized by using a neutral comet assay (Cat #4250-050-K, Trevigen, Gaithersburg, MD). Cells were plated on 10-cm BD Falcon Cell Culture Plates (BD, Franklin Lakes, NJ), incubated for 2 days, treated with 10 *μ*M MP470 or dimethylsulfoxide (control) for 1 hour, and then irradiated with 8 Gy. Cells were then trypsinized, placed on glass slides, and subjected to electrophoresis according to the manufacturer's instructions. dsDNA breaks were measured by olive tail movement, (OTM), defined as (tail length) × (the fraction of total DNA in the tail)[13]. OTM values were calculated with TriTek Comet Score V 1.5 software (The TriTek Corporation, Sumerduck, VA). Data points represent means ± SDs from triplicate experiments.

### Western blotting

Cells were plated on 10-cm petri dishes and grown for 24-48 hours. MP470 was then added at a concentration of 10 *μ*M (in 100% dimethlysulfoxide) for maximum inhibition. Cells were incubated with the MP470 for 24 hours (unless otherwise specified) before being irradiated with 4 Gy. After irradiation, cells were lysed on the plates by adding 350 *μ*L of sodium dodecyl sulfate lysis buffer (20 mM HEPES [pH 7.9], 400 mM NaCl, 1 mM EDTA, 1 mM EGTA, 1% NP40, 1 mM DTT, 1 mM phenylmethylsulfonyl fluoride, 1 mg/mL aprotinin, 1 mg/mL leupeptin, 250 mg/mL benzamide, 50 mM NaF, and 1 mM NaO_3_V_4_). The lysate was transferred to a 1.5-mL microcentrifuge tube, boiled for 5 minutes with intermittent vortexing, and then centrifuged for 5 minutes at 10,000 rpm, after which the supernatant was transferred to a new microcentrifuge tube. Lysates were subjected to electrophoresis on 10%-20% HCl pre-poured gels (Cat #161-1108, Bio-Rad, Hercules, CA). The proteins were then transferred to nitrocellulose paper and probed with the appropriate antibodies under the conditions recommended by the suppliers. The following antibodies were used Phospho-AKT (Ser 473 Cat, #4058), glycogen synthase kinase (GSK)-3β with Phospho-GSK-3β (Ser9, #9336) Cell Signaling Technology, Danvers, MA), RAD51 H-92 (Santa Cruz Biotechnology, Santa Cruz, CA) and c-Met phosphospecific Anti-c-Met [pY^1003^] (Cell Signaling Technology) [14].

### siRNA experiments

siRNA to c-Met (sc-29397) and control siRNA (sc-37007) were purchased from Santa Cruz Biotechnology. The transfection reagent Lipofectamine was from Invitrogen (Carlsbad, CA). U87 cells were grown to 70% confluence and transfected with siRNA at a final concentration of 100 nM. Seventy-two hours later, the cells were lysed for western blotting analysis as described above.

### Animal experiments

To create subcutaneous tumors, cells (4 × 106 per animal) were implanted in the flanks of 32 outbred athymic nude mice, 8 per arm (Taconic, Germantown, NY). U87 cells were chosen for their high level of c-Met expression and ability to rapidly produce tumors. Twenty-five days after the cells were injected, animals were pair-matched (based on tumor volume) and assigned to one of four treatment groups: control; MP470 (60 mg/kg) alone; radiation alone (2 Gy per day × 10 days); and MP470 + radiation. MP470 was delivered daily by gavage at a dose of 60 mg/kg in peanut oil starting on day 25 for 14 consecutive days. Radiation was started on day 27 and consisted of 2 Gy per day delivered to the tumor by a cobalt-60 irradiator (Theratron-80).

Radiation was delivered daily, 5 days per week for 2 weeks, at 1 hour after the MP470 treatment. The total cumulative dose delivered to the tumor was thus 20 Gy. Animals were euthanized by CO_2 _asphyxiation when the tumor volume reached 2000 mm^3^, as required by our institutional animal care and use committee protocol #07-029. All remaining animals were euthanized on day 48. Tumors were measured with calipers every 5 days and the volume calculated according to the formula (a^2 ^- b/2), where a is the smallest diameter and b is the largest diameter of the tumor.

Tumor growth delay was expressed in absolute and normalized terms as follows. Absolute growth delay (AGD) was defined as the number of days for tumors in the radiation-only and the MP470 + radiation groups to reach 1,500 mm^3 ^minus the number of days for tumors in the control group to reach the same size. Normalized growth delay (NGD) was calculated as the number of days for tumors in the combined-therapy group to reach 1,500 mm^3 ^minus the number of days for tumors in the MP470-only group to reach 1,500 mm^3^. The enhancement factor was then determined by dividing the NGD for the group receiving MP470 plus radiation by the AGD for the group given radiation alone. All statistical analyses were carried out with Stata 9.2 for Windows (College Station, TX), and *P *values < 0.05 were considered significant.

## Results

### Cytotoxicity and radiosensitization

**Figure 1:**
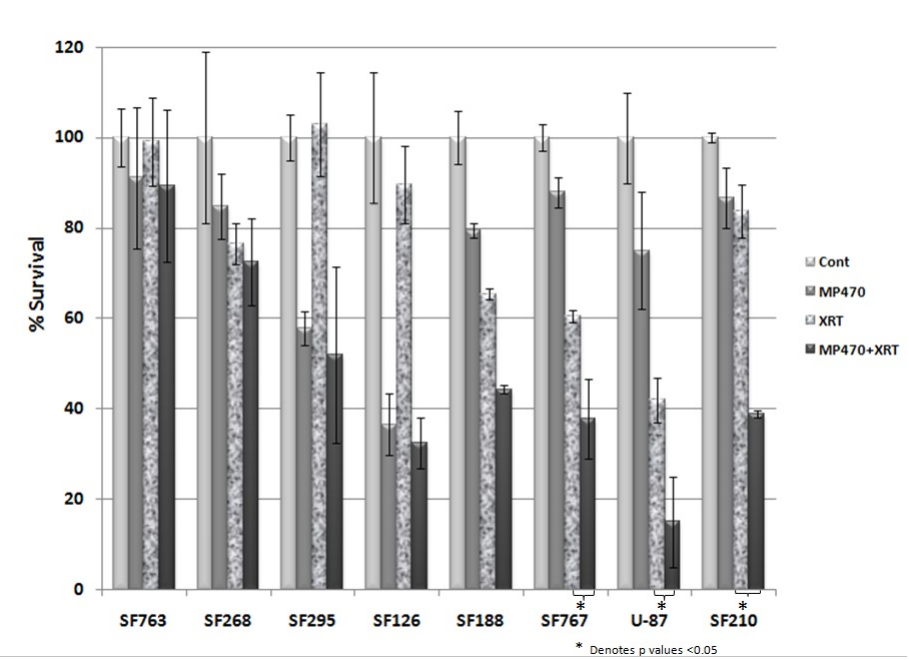
**Effect of MP470 and radiation on clonogenic survival of eight GBM cell lines**. The combination of MP470 (5 μM) plus radiation (4 Gy) produced the most cell killing of any treatment condition, but the extent of the responses varied among the different cell lines. The radiosensitizing effect of MP470 was most effective in the SF767, U87, and SF210 cell lines. Each bar is the mean of 3 experiments and error bars indicate standard deviation.

**Figure 2:**
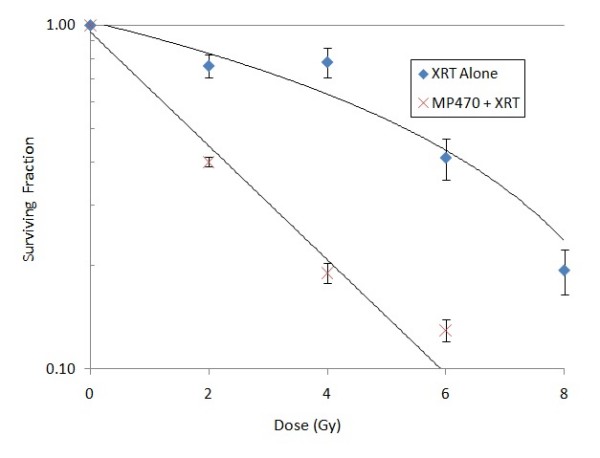
**Clonogenic survival of SF767 cells treated with MP470 followed by irradiation in a range of radiation doses**. Radiation at all doses had a moderate suppressive effect on clonogenic survival, and that effect was enhanced by pretreatment with MP470. Each data point represents the mean of 3 experiments ± SD.

The small-molecule tyrosine kinase inhibitor MP470 was designed to target c-Met, although it also inhibits the c-Kit receptor and platelet-derived growth factor receptor at nanomolar levels [11]. To evaluate its effect on proliferation eight GBM cell lines were used in an MTS assay. All eight cell lines proved to be sensitive to MP470 alone, with IC_50 _values ranging from 1 *μ*M to 10 *μ*M (median 5 *μ*M). To test its potential as a radiosensitizer, we assessed clonogenic survival after 4 Gy of the same eight GBM cell lines after a 1-hour treatment with MP470 followed by a single radiation dose (Fig. 1). Various levels of response were seen in the different cell lines, with 3 of the 8 GBM lines appearing to have a greater then additive response when MP470 was combined with XRT. SF767 cells were chosen to assesses for clonogenic survival in response to increasing doses of radiation (0 to 8 Gy) and MP470 had a radiosensitizing effect at all radiation doses tested, MP470 increased cell kill by 0.5 log compared to 4 Gy alone (Fig. 2).

### MP470 inhibits GSK3β and induces apoptosis

**Figure 3:**
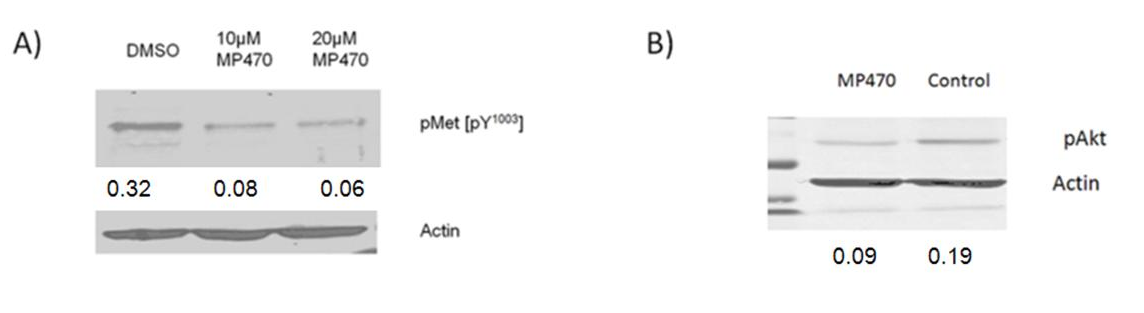
**Western blot analysis of SF767 cell lines in response to MP470**. (A) Treating SF767 cells with MP470 (10 *μ*M) for 1 hr followed by stimulation with pervanadate led to reduced phosphorylation of c-Met [pY^1003^] in a dose-dependent manner, compared to DMSO control. (B) SF767 cells treated with MP470 (10 *μ*M) for 1 hr showed reduced expression of pAkt protein.

**Figure 4:**
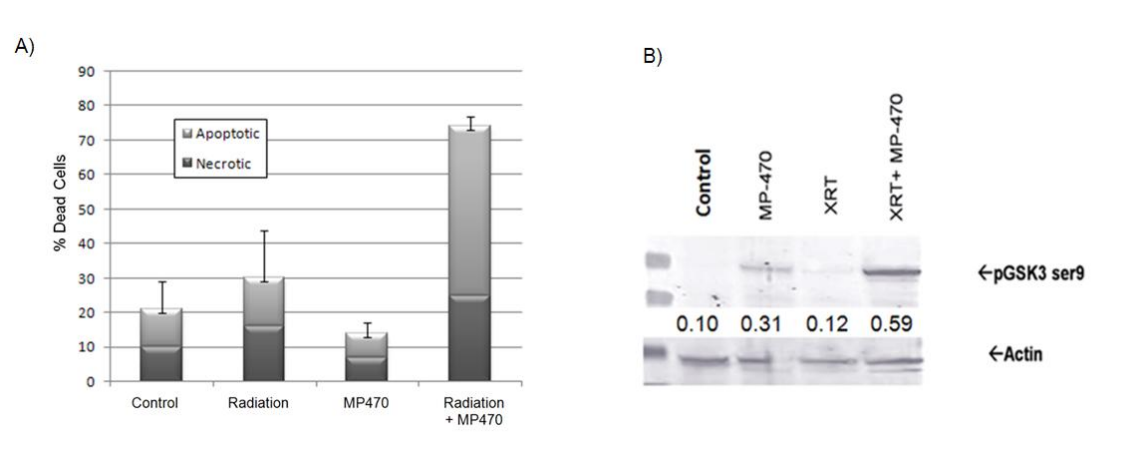
**(A) Apoptosis and necrosis, quantified using an acridine orange assay, in SF767 cells after treatment with radiation, MP470, or both. MP470 (5 *μ*M) alone had no effect on the proportion of dead cells**. Radiation (8 Gy) by itself induced some necrotic cell death but little apoptosis. However, the combination of MP470 followed by radiation greatly increased the proportions of both necrotic and apoptotic cells. Performed in triplicate: bars indicate mean ± SD. (B) Western blot analysis of SF767 cells treated with radiation (4 Gy) alone had no influence on the expression of glycogen synthase kinase (GSK)3β phosphorylated at serine 9; however, treatment with MP470 (10 *μ*M) or MP470 for 24 hrs followed by radiation enhanced the expression of this phosphorylated protein.

Having established the ability of MP470 to sensitize GBM cells to radiation, we next wanted to validate that it was acting through c-Met. SF767 cells demonstrate the presence of pMet and treatment with MP470 reduced c-Met phosphorylation (Fig. 3A), as assessed by immunoblotting analysis. In order to confirm MP470's mechanism of action we evaluated a known downstream pathway of c-Met, phosphatidylinositol 3-kinase/Akt, in SF767 cells [15]. A 1-hour incubation with MP470 led to a reduction in pAkt protein in SF767 cells (Fig. 3B). To determine the effect of this reduction in pAkt on cell survival, we evaluated apoptosis and necrosis induced by radiation (8 Gy), alone or after a 1-hour pretreatment with MP470, using an acridine orange assay. MP470 alone had no effect on cell death, and radiation alone induced a mild increase in cell death (this stands in contrast to the percent of death seen in a clonogenic assay, since the majority of radiation induced cell death is from mitotic division and not apoptotic). The combination of MP470 followed by radiation (8 Gy), however, killed 75% of the cells (Fig. 4A). We next postulated that GSK3β, a key regulator of the extrinsic apoptotic pathway [16], could play a role in this induction of apoptosis, as it is strongly regulated by Akt. We found that pretreatment with MP470 resulted in increased phosphorylation of GSK3β at serine-9 (Fig. 4B), a site known to inhibit GSK3β [16].

### Influence of MP470 on repair of dsDNA breaks

**Figure 5:**
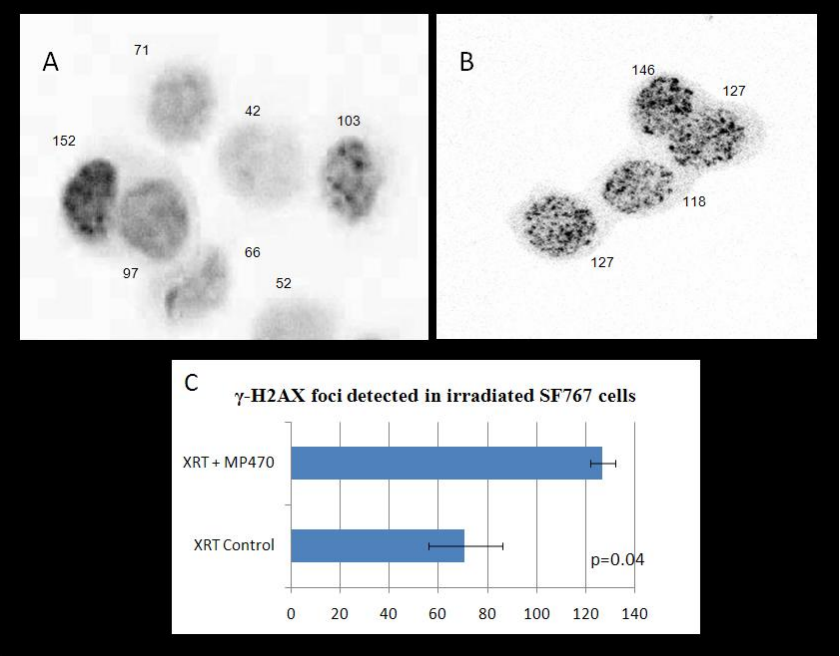
**Photomicrographs of γ-H2AX foci detected in irradiated SF767 cells**. Cells were irradiated with 4 Gy, fixed 8 hours later, and then stained with anti-γ-H2AX antibodies. Original magnification × 1,000 (values indicate mean intensity, based on densitometry). (A) γ-H2AX foci in SF767 cells treated with radiation alone. (B) Pretreatment with 5 *μ*M MP470, before radiation lead to increased γ-H2AX foci. (C) Radiation plus MP470 resulted in a median γ-H2AX foci intensity of 127 compared to 71 for radiation alone p = 0.04.

**Figure 6:**
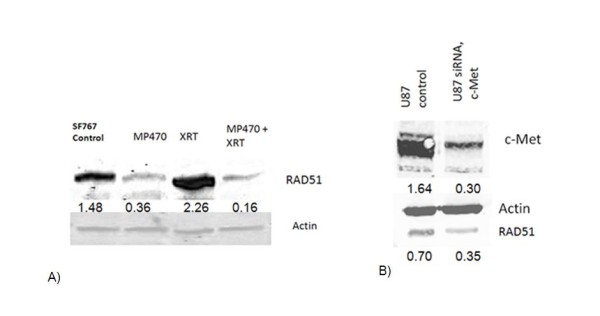
**(A) Western blot analysis of SF767 cells showed constitutive expression of the RAD51 which is elevated further after irradiation**. MP470 (10 *μ*M) inhibited both constitutive and radiation-induced expression of RAD51 protein. (B) Cells treated with siRNA to c-Met for 48 hours led to suppression of c-Met and reduced expression of RAD51.

To test the hypothesis that MP470 enhances radiation-induced cell death by influencing the repair of dsDNA breaks, we measured levels of γ-H2AX. At 1 hour after irradiation, both the control cells and the MP470-treated cells showed comparable numbers of γ-H2AX foci, suggesting that MP470 does not enhance the initial level of radiation induced dsDNA breaks. In order to detect an influence of MP470 on repair, we quantified the level of γ-H2AX foci several hours after irradiation. At 8 hours after irradiation, cells treated with XRT had a median densitometry intensity of 71 compared to 127 for cells treated with MP470 and XRT p = 0.04. (Fig. 5). To further evaluate MP470's affect on dsDNA repair, we supplemented our γ-H2AX results with a comet assay. At 1 hour after irradiation, SF767 cells treated with either radiation alone (8 Gy) or with 10 *μ*M MP470 followed by irradiation showed similar levels of DNA damage, higher doses of MP470 and radiation were used here due to the low sensitivity of the comet assay. However, at 8 hours after irradiation, dsDNA repair was greatly inhibited in the cells that had been pretreated with MP470 22 ± 3.1 tail DNA (%), for 8-Gy irradiation alone and 35 ± 4.3 tail DNA (%), for MP470 followed by 8-Gy irradiation). This increase in OTM suggests that MP470's radiosensitizing effect may be partially mediated through inhibition of dsDNA repair. RAD51 is a key regulator of homologous recombinational repair [17,18] and our prior work has demonstrated that RAD51 level at the time of surgical resection is an independent prognosticator of survival in GBM patients [19], thus we evaluated whether MP470 could affect RAD51. RAD51 expression was noted to be increased (24 hrs) after the cells were irradiated. Pretreatment with MP470 decreased RAD51 expression in nonirradiated cells and suppressed the increase in expression prompted by radiation (Fig. 6A). This effect was dose-dependent, with the strongest suppression at MP470 concentrations exceeding 5 *μ*M. To verify that MP470 was indeed decreasing RAD51 expression and not simply shifting cells into a quiescent cell cycle state (G_0_) characterized by lower levels of RAD51, we tested the effect of MP470 on cell cycle distribution and found it had no influence. To establish that RAD51 suppression was directly associated with c-Met inhibition, we silenced c-Met expression using siRNA, which also demonstrated inhibition of RAD51 (Fig. 6B).

### In vivo radiosensitization studies

**Figure 7:**
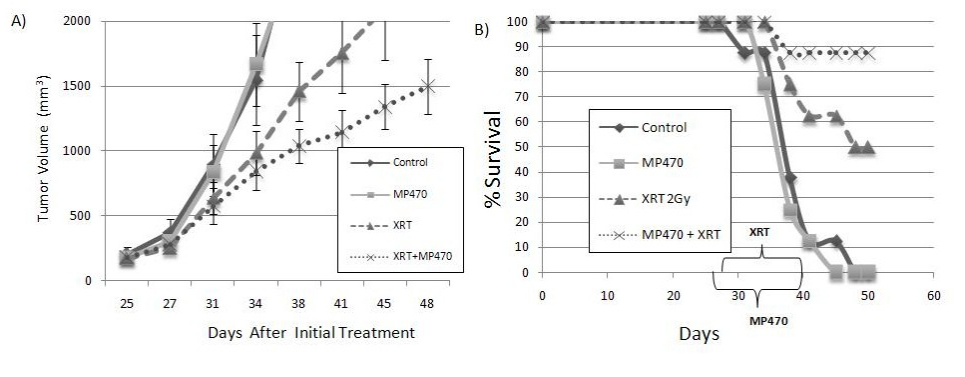
**Tumor volume and survival in mice with subcutaneous U87 MG xenografts treated with MP470 and radiation**. Tumor volume of 1,500 mm^3 ^was used as a baseline for calculating tumor growth delays, and mice were euthanized once they reached 2000 mm^3^. (A) Irradiation alone resulted in a modest AGD of 6.1 ± 2.3 days, while the combination of MP470 plus irradiation produced an AGD of 17.7 ± 2.8 days, resulting in an enhancement ratio of 2.9 (Table 1). (B) Kaplan-Meier survival rates on day 48 (the final day of the experiment) were 0% in both the control and MP470-alone groups, 50% in the radiation-only group (*P *= 0.035), and 87.5% in the MP470 + radiation group (*P *= 0.001). Each data point represents the mean of 8 tumors; bars indicate standard error.

To validate the *in vitro *results, we implanted GBM cells subcutaneously in the flanks of nude mice and treated those mice with MP470, irradiation, or both, with 8 animals per group. Treatment started on day 25 with MP470 which was given daily for 14 consecutive days, XRT was started on day 27 using a total of 20 Gy in 10 daily fractions, to the tumor alone. On day 48 after implantation the experiment was terminated and the tumors were measured. As shown in Fig. 7A, MP470 increased the AGD from 6.1 ± 2.3 days with radiation alone to 17.7 ± 2.8 days with the combination, resulting in an enhancement ratio of 2.9 (Table 1). Survival rates were evaluated on the final day of the experiment. At that time, survival rates were 0% in the vehicle-control or MP470-only groups, 50% in the radiation-only group (*P *= 0.035), and 87.5% in the MP470-plus-radiation group (*P *= 0.001) (Fig. 7B).

**Table 1:** MP470-induced tumor growth delay in U87 xenografts

Treatment Group	No. of Mice Per Group	Tumor Growth Period, days*	**Absolute Growth Delay**^†^	**Normalized Growth Delay**^‡^	Enhancement Factor**
Control	8	9.7 ± 1.01			
MP470	8	8.6 ± 1.1	0 ± 1.1		
Radiation	8	15.8 ± 2.3	6.1 ± 2.3		
MP470 + Radiation	8	27.4 ± 2.8	17.7 ± 2.8	17.7 ± 2.8	2.9

* Time for subcutaneous tumors to grow from 200 mm^3 ^to 1500 mm^3 ^(see text).

^† ^No. of days for tumors in the radiation-only and the MP470 + radiation groups to reach 1,500 mm^3 ^minus no. of days for tumors in the control group to reach the same size.

^‡ ^No. of days for tumors in the MP470 + radiation group to reach 1,500 mm^3 ^minus no. of days for tumors in the MP470-only group to reach the same size.

** Normalized growth delay for MP470 + radiation group divided by absolute growth delay for the radiation-only group.

## Discussion

The small molecule MP470 is a potent c-Met antagonist that is cytotoxic to a variety of cell lines *in vitro *[11]. In this report, we demonstrated that concurrent inhibition of c-Met in combination with irradiation led to both reduced dsDNA repair and enhanced apoptosis in GBM. Our *in vitro *findings were supported by our *in vivo *observations using a xenograft model in nude mice. In this model, MP470 by itself, at a dose of 60 mg/kg, had no effect on tumor size or survival; radiation by itself was somewhat more effective in reducing tumor volume and improving survival; but the combination of radiation plus MP470 produced the best response in terms of both local control and survival.

High-grade glial neoplasms of the brain continue to be one of the most challenging malignancies to treat, and their poor prognosis has improved only marginally over the past four decades (survival rates of 10%-20% at 2 years) [20]. Postoperative radiation provides a clear survival advantage for patients with gliomas, yet the majority of disease recurrences present within 2 cm of the postoperative bed-the very place targeted by the radiation. Unfortunately, attempts to escalate treatment doses to the tumor bed have provided only modest benefit [21]. To better understand why requires evaluating the cellular and molecular interactions in the resistant tumor cells. The pathway to malignancy consists of multiple genetic mutations, often in key regulators of the cell cycle or DNA repair process. These alterations allow cancer cells to not only divide unchecked, but also to repair DNA damage at an accelerated or more efficient rate. One of the genes implicated in homologous recombination repair of dsDNA damage is RAD51. Prior work from our lab has demonstrated that RAD51 expression levels at the time of initial surgical resection are an independent prognosticator of survival for GBM patients receiving radiation [19]. In the present paper, we evaluated whether MP470 could influence RAD51 expression in GBM tumors cell and found that pretreatment with MP470 inhibited XRT induced expression of RAD51. This compliments our prior GBM tissue microarray findings that 70% of recurrent GBM tumors, treated with XRT, were found to have elevated RAD51 at the time of recurrence [19]. Paradoxically, this suggests that the ability of malignant cells to repair dsDNA damage can be enhanced by the very agents used to treat malignancies. The stimulation of RAD51 by radiation may explain why current therapies temporarily improve local control but fail to offer definitive cures. Clearly, substantial improvements in local control and survival of patients with GBM will require targeting (inhibiting) the molecular machinery that mediates the development of resistance.

To our knowledge, this is the first demonstration that MP470, an orally available c-Met antagonist, causes radiosensitization of several GBM cell lines. We have shown evidence that supports a mechanism of action consistent with a decrease in dsDNA break repair, along with enhanced radiation-induced apoptosis. Other investigators have shown that c-Met inhibition can enhance radiation-induced tumor cell death *in vitro *using a retrovirally based approach that would not be a clinically viable option, although it did serve as an important proof of concept [10]. This stands in contrast to MP470, which is well tolerated in animals, with no observable adverse effects from daily administration of 2,000 mg/kg to rats and 240 mg/kg to dogs [22]. This initial work on MP470 provided the foundation to support a phase I trial, to establish the maximum tolerated dose of MP470 in humans. Our work reported here suggests that c-Met inhibition can offer therapeutically relevant radiosensitization and potentially improve the therapeutic ratio in radiation-resistant tumors such as GBM.

## Competing interests

James Welsh, Research support from SuperGen, holds patent on MP-470

Daruka Mahadevan, Co-discovered of MP470, holds patent on MP-470

David Bearss, Co-discovered of MP-470 and employee of Surgen, holds patent on MP-470

All other authors have no conflicts of interest.

## Authors' contributions

JW developed the ideas for these experiments, performed much of the work, and drafted the manuscript. DM was involved with experimental design and provided resource to perform this work. RE performed some of the experiments. LC provided technical laboratory support. DB provided resource and technical advice. BS provided resources and participated in the design and coordination. All authors read and approved the final manuscript.
